# Synthesis, Characterization and Antitumor Mechanism Investigation of Heterometallic Ru(Ⅱ)-Re(Ⅰ) Complexes

**DOI:** 10.3389/fchem.2022.890925

**Published:** 2022-05-27

**Authors:** Xiurong Ma, Junjian Lu, Peixin Yang, Bo Huang, Rongtao Li, Ruirong Ye

**Affiliations:** ^1^ Faculty of Life Science and Technology, Kunming University of Science and Technology, Kunming, China; ^2^ Faculty of Chemistry and Chemical Engineering, Yunnan Normal University, Kunming, China

**Keywords:** heteronuclear metal complexes, ruthenium(II) complexes, rhenium(I) complexes, anticancer activity, apoptosis

## Abstract

The development of heteronuclear metal complexes as potent anticancer agents has received increasing attention in recent years. In this study, two new heteronuclear Ru(Ⅱ)-Re(Ⅰ) metal complexes, [Ru(bpy)_2_LRe(CO)_3_(DIP)](PF_6_)_3_ and [Ru(phen)_2_LRe(CO)_3_(DIP)](PF_6_)_3_ [**RuRe-1** and **RuRe-2**, L = 2-(4-pyridinyl)imidazolio[4,5-f][1,10]phenanthroline, bpy = 2,2′-bipyridine, DIP = 4,7-diphenyl-1,10-phenanthroline, phen = 1,10-phenanthroline], were synthesized and characterized. Cytotoxicity assay shows that **RuRe-1** and **RuRe-2** exhibit higher anticancer activity than cisplatin, and exist certain selectivity toward human cancer cells over normal cells. The anticancer mechanistic studies reveal that **RuRe-1** and **RuRe-2** can induce apoptosis through the regulation of cell cycle, depolarization of mitochondrial membrane potential (MMP), elevation of intracellular reactive oxygen species (ROS), and caspase cascade. Moreover, **RuRe-1** and **RuRe-2** can effectively inhibit cell migration and colony formation. Taken together, heteronuclear Ru(Ⅱ)-Re(Ⅰ) metal complexes possess the prospect of developing new anticancer agents with high efficacy.

## Introduction

Cancer is a malignant disease that seriously threatens human health and life (Lortet-Tieulent et al., 2020). Although platinum-based drugs show outstanding antitumor activity, they also exhibit strong toxic side effects and drug resistance (Oun et al., 2018). This makes the development of non-platinum drugs particularly important. Ruthenium metal complexes, which offer the advantages of easy cellular uptake (Notaro and Gasser., 2017), good biodistribution (Sun et al., 2021), low toxicity (Thota et al., 2018), induction of cell apoptosis (Galczynska et al., 2020), selective anti-invasion and anti-metastasis activity (Cao et al., 2017), are the most promising non-platinum antitumor drugs. At present, several ruthenium complexes have entered clinical studies, including NAMI-A (Alessio and Messori, 2019), KP1019 (Alessio and Messori, 2018), KP1339 (Heffeter et al., 2013), TLD1433 (McFarland et al., 2020). Ruthenium complexes can exert their anti-tumor activity by interacting with biomolecules such as proteins (Nehru et al., 2020; da Silva et al., 2021), DNA (Gill and Thomas, 2012; Li et al., 2016; Zhang et al., 2018), RNA (Jain et al., 2013; Wang P. et al., 2021), and subcellular organelles (Qiu et al., 2017; Huang et al., 2020; Tan et al., 2021). For example, Ru(II) polypyridine complexes containing the planar ligand DPPZ (dipyrido[3,2-a:2′,3′-c]phenazine) show high insertion affinity with DNA and act as the “light switch” of DNA molecules (Holmlin et al., 1998). By coupling Ru(II)-polypyridyl moiety with a phenanthroline substituted SAHA (suberoylanilide hydroxamic acid) derivative, our group reported three Ru(II)-based histone deacetylases inhibitors that show excellent antitumor activity and histone deacetylase inhibition (Ye et al., 2013). In summary, ruthenium-based metal complexes play an important role in the development of antitumor drugs. Liu’s group reported a series of organometallic half-sandwich Ru(II) complexes bearing aryl-BIAN chelating ligands that can elicit cytotoxicity through lysosome-mediated apoptosis *in vitro* and suppress tumor growth *in vivo* (Xu et al., 2020).

In addition to ruthenium complexes, rhenium complexes also exhibit potent anticancer activity and have attracted much attention in metal-based anticancer drugs (Leonidova and Gasser, 2014; Collery et al., 2019; Capper et al., 2020). Rhenium-based compounds exhibit high stability (Kumar et al., 2016), structural diversity (Imstepf et al., 2016), ease of real-time imaging (Palmioli et al., 2017) and lack of off-site toxicity (Capper et al., 2020). Recently, by integrating deferasirox with Re(I) moiety, Mao’s group designed a mitochondria-targeted rhenium(I) complex that disrupts both mitochondrial metabolism and iron homeostasis (Pan et al., 2020). Phosphorescent Re(I) tricarbonyl complexes bearing β-carboline derivatives exhibit pH-dependent phosphorescence that specifically image lysosomes, causing lysosomal dysfunction and impaired lysosomal activity, which in turn leads to autophagy and apoptosis-dependent cell death (He et al., 2019). Our group reported a series of phosphorescent rhenium(I) complexes conjugated with artesunate, showing mitochondrial targeting and dual induction of apoptosis-ferroptosis (Ye et al., 2021). The photodynamic anticancer activity of Re(I) complexes has also been extensively studied (Leonidova et al., 2014; Liew et al., 2020; He et al., 2022).

Heterobimetallic complexes have been explored in the anticancer field in order to associate different metals within a single entity to enhance their activity (Jain, 2019; Johnson et al., 2020; Guedes et al., 2020; Tsolis et al., 2021). Pt(II)-Re(I) complexes synthesized by Paulo’s team showed dual imaging and anticancer properties (Quental et al., 2017). Compared with Pt(II)-Re(I) heteronuclear metal complexes, the antitumor properties of Pt(II)-Ru(II) heteronuclear metal complexes are relatively more studied. Pt(II)-Ru(II) complexes reported by singh’s group could bind to DNA and showed phototoxicity to MCF-7 cells (Singh et al., 2021). Brenda S. J. Winkel’s group reported that the polyazine bridged Pt(II)-Ru(II) complex displayed significant DNA modification, cell growth inhibition, and toxicity towards F98 malignant glioma cells following visible light irradiation (Zhu et al., 2017). Pt(II)-Ru(II) complex designed by Mao and Tan’s team could overcome cisplatin resistance by photodamaging mitochondrial DNA (Zheng et al., 2018). The research on Ru(II)-Re(II) heteronuclear metal complexes mostly focus on catalysis (Coleman et al., 2008; Li et al., 2021). There are few publications reporting the DNA switching (Foxon et al., 2007; Jarman et al., 2019) and pH luminescence switching (Zheng et al., 2014) effects of these complexes.

**SCHEME 1:**
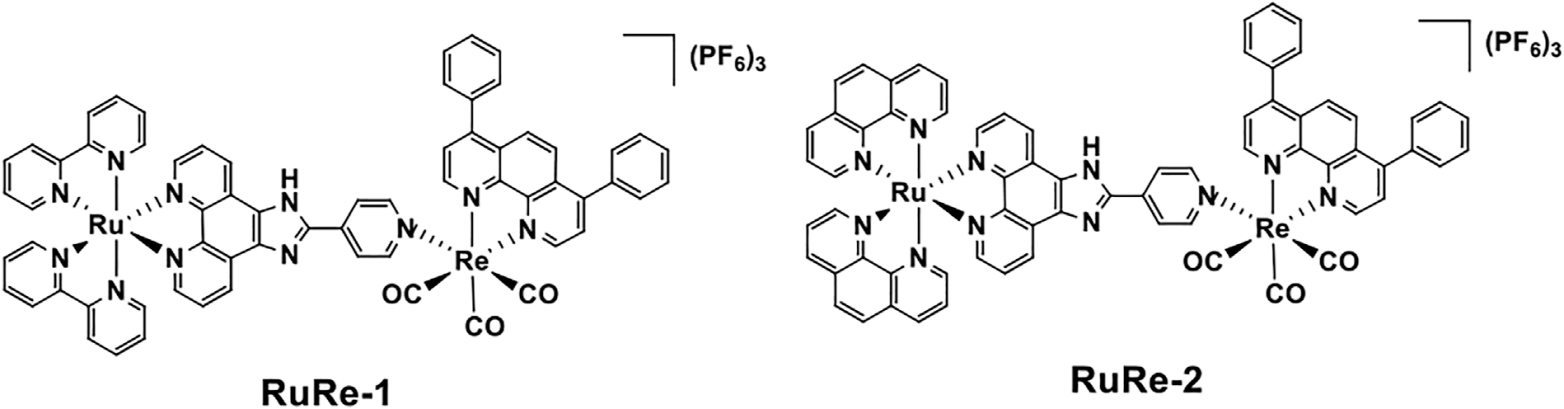
Chemical structures of **RuRe-1** and **RuRe-2**.

In this context, two heterobimetallic complexes based on Ru(II) and Re(I) units, [Ru(bpy)_2_LRe(CO)_3_(DIP)](PF_6_)_3_ and [Ru(phen)_2_LRe(CO)_3_(DIP)](PF_6_)_3_ (**RuRe-1** and **RuRe-2**, L = 2-(4-pyridinyl)imidazolio[4,5-f][1,10]phenanthroline, bpy = 2,2′-bipyridine, DIP = 4,7-diphenyl-1,10-phenanthroline, phen = 1,10-phenanthroline), were designed and synthesized (Scheme 1). We first evaluated their anti-proliferative activities. Then, the anticancer mechanisms of **RuRe-1** and **RuRe-2** were discussed in detail, including the effects on apoptosis, cell cycle, mitochondrial membrane potential (MMP), reactive oxygen species (ROS), cell migration and colony formation. These findings will contribute to the development of heteronuclear metal complexes in anticancer field.

## Results and Discussion

### Synthesis, Characterization, and Photophysical Properties

The synthetic routes of **RuRe-1** and **RuRe-2** are shown in Supplementary Figure S1. Firstly, mononuclear complexes **Ru-1** (Halpin et al., 2013) and **Re-1** (Woźna and Kapturkiewicz, 2015) were synthesized according to literature methods. **Ru-2** was prepared following a similar procedure to that of **Ru-1**, except that *cis*-[Ru(phen)_2_Cl_2_]·2H_2_O (Sullivan et al., 1978) was used instead of *cis*-[Ru(bpy)_2_Cl_2_]·2H_2_O (Hartshorn and Barton, 1992). Target complexes **RuRe-1** and **RuRe-2** could be successfully synthesized through the direct reaction of **Ru-1** or **Ru-2** with **Re-1** in acetone. After most of the solvents were concentrated in vacuum, a red precipitate was obtained by dropwise addition of saturated NH_4_PF_6_ aqueous solution, then the crude product was purified by silica gel column chromatography with a mobile phase of acetonitrile: water: saturated potassium nitrate = 100: 9: 1. The products were characterized using ESI-MS, ^1^H NMR, FT-IR (Supplementary Figures S2–S9) and elemental analysis.

The electronic absorption and emission spectra of mononuclear complexes (**Ru-1**, **Ru-2**, **Re-1**) and heteronuclear **RuRe-1** and **RuRe-2** were recorded in phosphate buffered saline (PBS), dichloromethane and acetonitrile at 298 K (Supplementary Figure S10 and Supplementary Figure S11). As shown in Supplementary Figure S11A, an intense absorption band at approximately 260–320 nm was observed, which could be assigned to the intraligand transition, and another two less intense absorptions in visible light range at approximately 350–400 nm and 420–500 nm could be ascribed to d(Ru)→L(π*) and d(Re)→L(π*) metal-to-ligand charge-transfer, respectively. Upon excitation at 455 nm, **RuRe-1** and **RuRe-2** showed similar emission bands, with the maximum emission around 590 nm in PBS (Supplementary Figure S11B). And the emission quantum yields (*Ф*_em_) of mononuclear complexes (**Ru-1**, **Ru-2**, **Re-1**) and heteronuclear **RuRe-1** and **RuRe-2** have also been determined. The *Ф*_em_ of **RuRe-1** and **RuRe-2** were similar to those of **Ru-1** and **Ru-2**. The photophysical data were summarized in Supplementary Table S1.

### Stability

The stabilities of **RuRe-1** and **RuRe-2** in PBS and human serum albumin (HSA) were tested through UV–Vis spectroscopy. As shown in Supplementary Figure S12, there was no significant change in the spectral characteristics and absorption peaks of **RuRe-1** and **RuRe-2** collected at 0, 24, and 48 h, indicating that these complexes are stable under physiological conditions.

### Lipophilicity and Cellular Uptake

Drugs exert their effects through pharmacokinetic processes such as absorption, distribution, and metabolism, which are closely related to the n-octanol/water partition coefficient of the drug (log *P*_o/w_) (Choi et al., 2012). Herein, the log *P*_o/w_ of **RuRe-1** and **RuRe-2** were determined by the shaking flask method to be 0.47 and 2.10, respectively, indicating that these two compounds are hydrophobic and can be well absorbed by cells.

**FIGURE 1:**
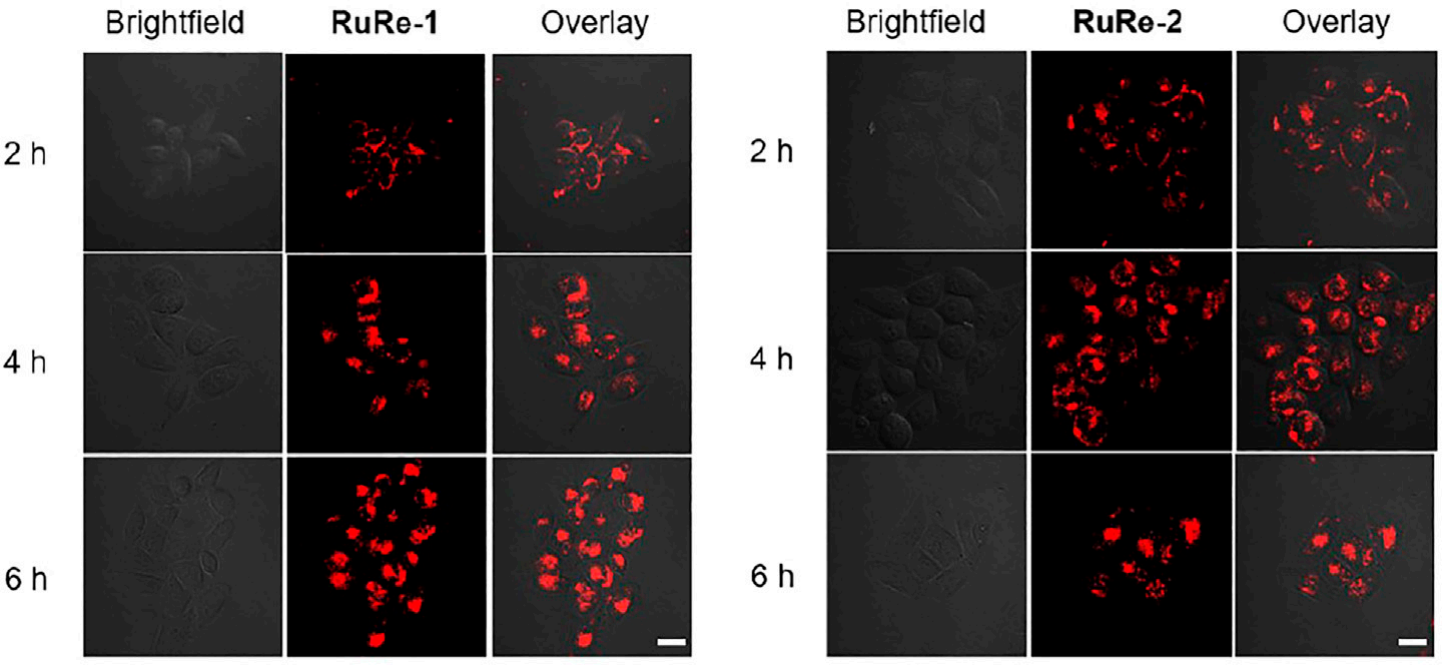
Intracellular uptake of **RuRe-1** (10 μM) and **RuRe-2** (10 μM) measured by confocal microscopy (*λ*_ex_ = 488 nm, *λ*_em_ = 590 ± 20 nm). Scale bar: 20 μm.

The cellular uptake of **RuRe-1** and **RuRe-2** was first qualitatively investigated by confocal microscopy. As shown in Figure 1, with the increase of incubation time, both complexes could effectively penetrate into HeLa cells and exhibited bright fluorescence in the cytoplasm. As exogenous elements, ruthenium and rhenium in cells can be quantified by inductively coupled plasma mass spectrometry (ICP-MS). Upon incubation with 10 μM **RuRe-1** and **RuRe-2** for 6 h, the ratio of intracellular ruthenium and rhenium content was approximately 1:1 (Supplementary Figure S13). This result further confirms the stability of the heteronuclear Ru(Ⅱ)-Re(Ⅰ) complexes under physiological conditions.

### *in vitro* Antitumor Activity

The anticancer activities of **RuRe-1** and **RuRe-2** against four cancer cell lines: HeLa (human cervical cancer cells), HepG2 (human hepatocellular carcinoma), A549 (human lung cancer cells), A549R (cisplatin-resistant A549) and one normal cell line LO2 (human normal liver cells) were determined by 3-(4,5-dimethylthiazole)-2,5-diphenyltetraazolium bromide (MTT) assay, while the mononuclear complexes **Ru-1**, **Ru-2**, **Re-1** and cisplatin were selected as controls. As shown in Table 1, **RuRe-1** and **RuRe-2** showed the best anticancer effects on HeLa cells, with IC_50_ values of 3.1 μM and 2.5 μM, respectively. And the *in vitro* antiproliferative efficacies of the compounds against HeLa cells were in the following order: **RuRe-2** > **RuRe-1** > **Re-1** > cisplatin > **Ru-1** > **Ru-2**. The mononuclear ruthenium complexes **Ru-1** and **Ru-2** showed negligible antitumor activity against all cancer cell lines screened. The anticancer activity of the mononuclear rhenium complex **Re-1** was between 5.6 μM and 8.0 μM, which contributed the most to the antitumor activities of the heteronuclear Ru(II)-Re(Ⅰ) metal complexes. In addition, **RuRe-1** and **RuRe-2** displayed approximately 8.7-fold and 5.4-fold greater ability to kill A549R cells than cisplatin, indicating that they can conquer the resistance of cisplatin. Furthermore, the cytotoxicity of **RuRe-1** and **RuRe-2** against LO2 cells was lower than that of HepG2 cells, revealing their selectivity to cancer cells.

**TABLE 1:** IC_50_ values of tested compounds towards different cell lines^a^.

Compounds	IC_50_ (μM)				
	HeLa	HepG2	A549	A549R	LO2
**RuRe-1**	3.1 ± 0.8	10.0 ± 0.8	11.8 ± 0.4	10.4 ± 1.2	25.1 ± 0.7
**RuRe-2**	2.5 ± 1.3	12.5 ± 0.9	11.2 ± 0.9	16.7 ± 0.8	28.1 ± 1.1
**Ru-1**	39.8 ± 0.6	>50	>50	>50	>50
**Ru-2**	43.6 ± 1.2	>50	>50	>50	>50
**Re-1**	7.7 ± 0.8	5.6 ± 0.5	6.9 ± 0.9	8.0 ± 1.1	5.8 ± 0.2
Cisplatin	19.3 ± 0.5	23.5 ± 2.0	25.9 ± 0.8	90.3 ± 0.3	22.5 ± 2.0

a IC_50_ values are drug concentrations necessary for 50% inhibition of cell viability. The data are presented as mean ± standard deviation (SD) and cell viability is assessed after 48 h of incubation.

### Apoptosis Assay

Apoptosis is an evolutionarily conserved form of programmed cell death that is essential for animal development and tissue homeostasis (Obeng, 2020). Apoptosis is characterized by a series of defined biochemical and morphological events, such as activation of caspase family proteases, loss of cell membrane asymmetry accompanied by phosphatidylserine translocation from the inner plasma membrane to the outer cell surface, cell shrinkage, nuclear fragmentation, chromatin condensation, and chromosomal DNA fragmentation.

**FIGURE 2:**
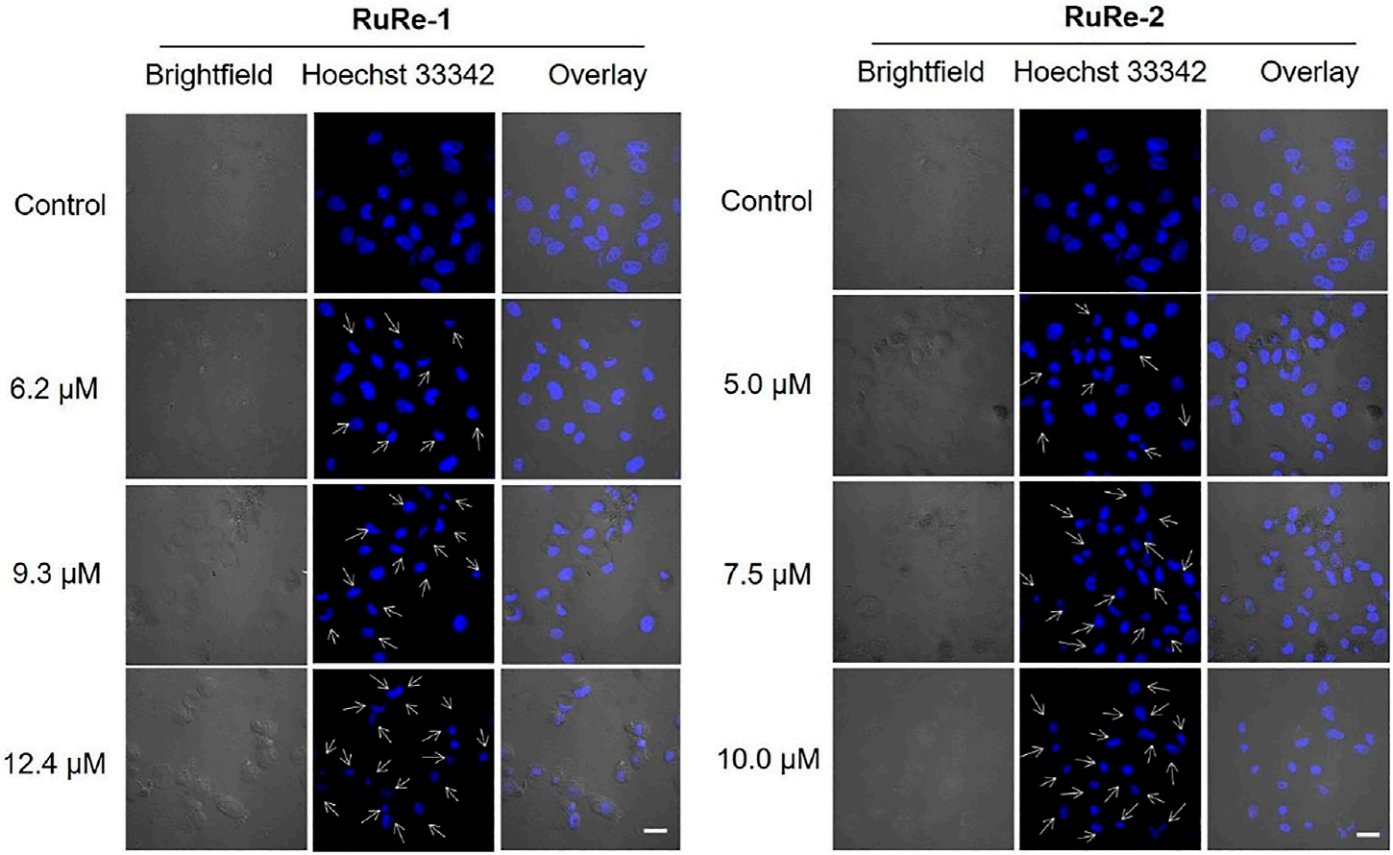
**RuRe-1** and **RuRe-2** induced HeLa cells apoptosis measured by confocal microscopy with Hoechst 33342 staining (*λ*_ex_ = 405 nm, *λ*_em_ = 460 ± 20 nm). The incubation time of the compounds was 24 h. Arrows indicate apoptotic morphological nuclei. Scale bar: 20 μm.

First, changes in cell morphology of HeLa cells induced by **RuRe-1** and **RuRe-2** were examined with 2'-(4-ethoxyphenyl)-5-(4-methyl-1-piperazinyl)-2,5′-bi-1H-benzimidazole trihydrochloride (Hoechst 33342) staining. Hoechst 33342 is a blue fluorescent dye that can penetrate cell membranes and is commonly used to stain cell nuclei (Tian et al., 2018). The results were given in Figure 2, the nuclei of the control group showed a regular round shape, while the cells treated with **RuRe-1** and **RuRe-2** showed typical apoptotic characteristics, in which the nuclei solidified into a homogeneous dense mass and then broke into fragments of different sizes, and the number of cells showing this phenomenon increased with the concentration of the drug administered.

**FIGURE 3:**
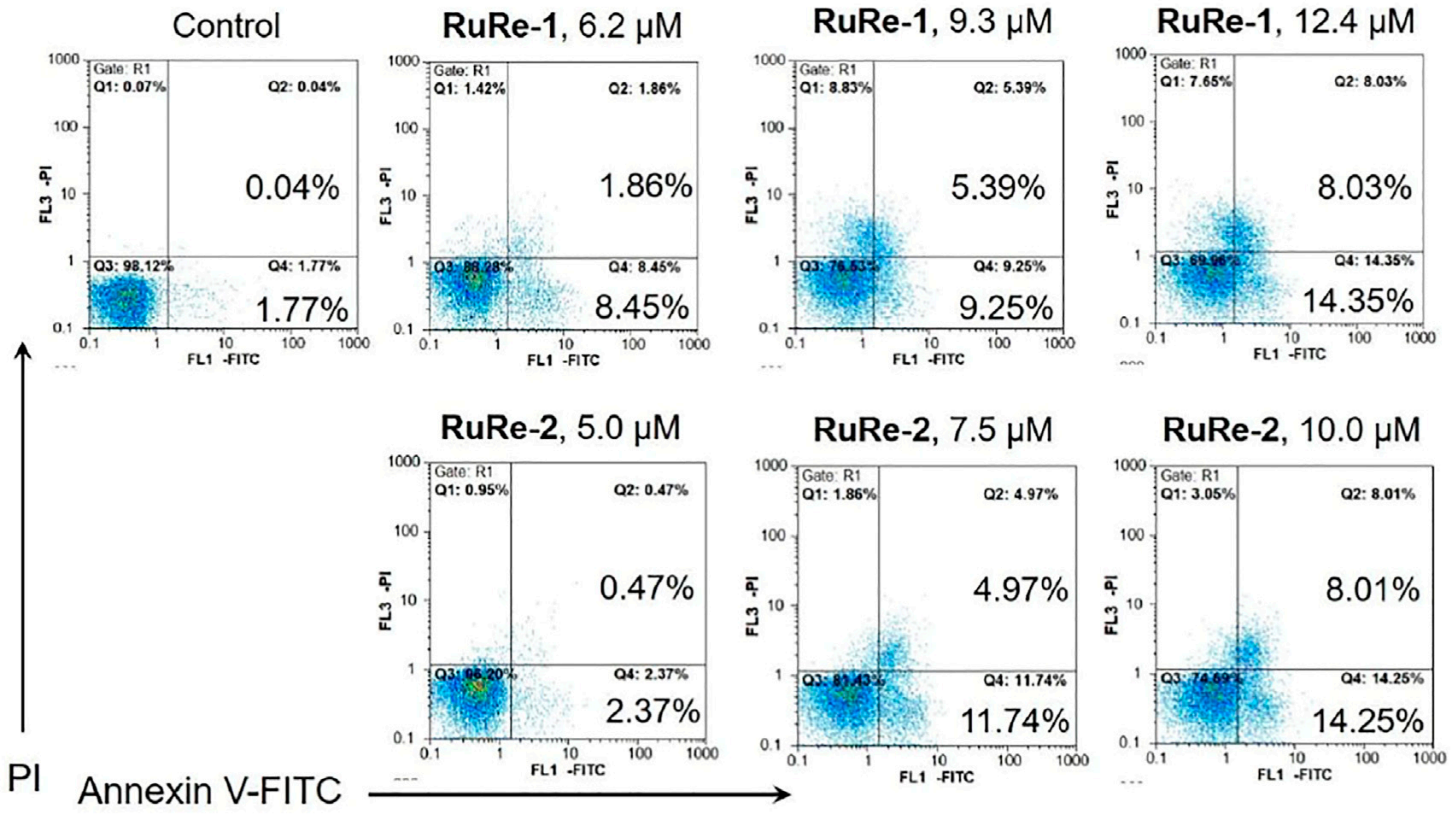
**RuRe-1** and **RuRe-2** induced HeLa cells apoptosis measured by flow cytometry with Annexin V/PI staining. The incubation time of the compounds was 24 h.

The ability of **RuRe-1** and **RuRe-2** to induce apoptosis in HeLa cells was further verified using Annexin V-FITC/PI (FITC: fluorescein isothiocyanate; PI: propidium iodide) double-staining. In the early stage of apoptosis, the surface of the cell membrane is broken, at which point the phosphatidylserine on the surface of the apoptotic cell flips from the inner cell membrane to the outer cell membrane, where it can be labelled by Annexin V (Jan and Chaudhry, 2019). The membrane permeability of PI is poor and thus only necrotic cells can be labelled (Zec et al., 2014). As shown in Figure 3, after treatment of cells with **RuRe-1** and **RuRe-2** for 24 h, the proportion of apoptotic cells (early apoptotic + late apoptotic) increased in a concentration-dependent manner. Specifically, treatment with **RuRe-1** (12.4 μM) or **RuRe-2** (10.0 μM) significantly increased the percentage of apoptotic cells from 1.81% (control) to 22.38% (**RuRe-1**) and 22.26% (**RuRe-2**), respectively. Overall, the results indicate their ability to induce apoptosis in HeLa cells.

**FIGURE 4:**
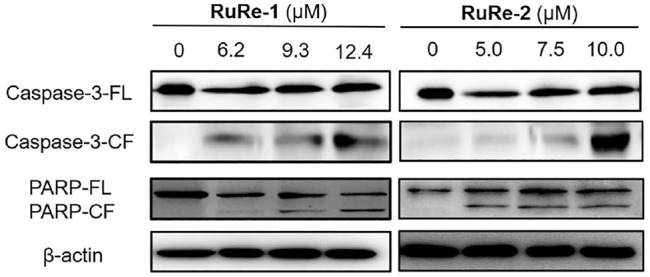
Expression of apoptosis-related protein (caspase-3 and PARP) in HeLa cells treated with **RuRe-1** and **RuRe-2** for 24 h.

Cysteine aspartate proteases play an important role in apoptosis, especially caspase-3 protein, which is one of the most important execution factors in the apoptotic pathway (Wang X. et al., 2021). The poly(ADP-ribose) polymerase (PARP) is associated with DNA repair and guardianship of genetic integrity (Pandey and Black, 2021). After treating HeLa cells with different concentrations of **RuRe-1** or **RuRe-2** for 24 h, the expression of caspase-3 and the caspase substrate PARP apoptotic protein were detected by western blot. As shown in Figure 4, **RuRe-1** and **RuRe-2** induced the cleavage of caspase-3 and PARP in a dose-dependent manner. It was further shown that **RuRe-1** and **RuRe-2** are activators of caspase-3, which can trigger apoptosis in HeLa cells.

**FIGURE 5:**
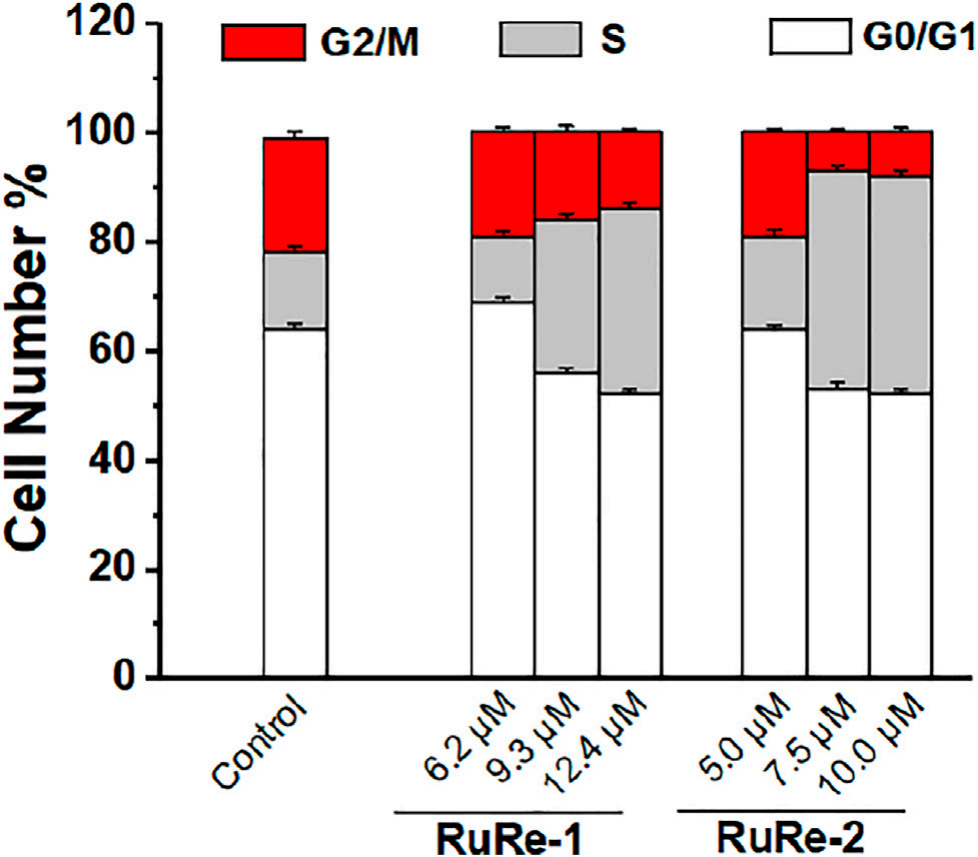
Quantitative cell-cycle distribution data for HeLa cells after treatment with **RuRe-1** and **RuRe-2** for 24 h.

Cell cycle arrest studies provide a good understanding of cell apoptosis induced by metal drugs (Sadoughi et al., 2021). To further clarify the mechanism of apoptosis induced by **RuRe-1** and **RuRe-2**, we analyzed the cell cycle of HeLa cells treated with **RuRe-1** and **RuRe-2** by flow cytometry with PI staining. The results (Figure 5, Supplementary Figure S14 and Supplementary Table S2) showed that compared with the control group, the content of S-phase cells in the **RuRe-1** (12.4 μM) and **RuRe-2** (10.0 μM) treated groups increased from 14.4 to 33.8% and 40.0%, respectively. A corresponding decrease in cell content in G1 and G2 phases was observed. The result suggests that **RuRe-1** and **RuRe-2** may induce cell death by regulating the cell cycle.

**FIGURE 6:**
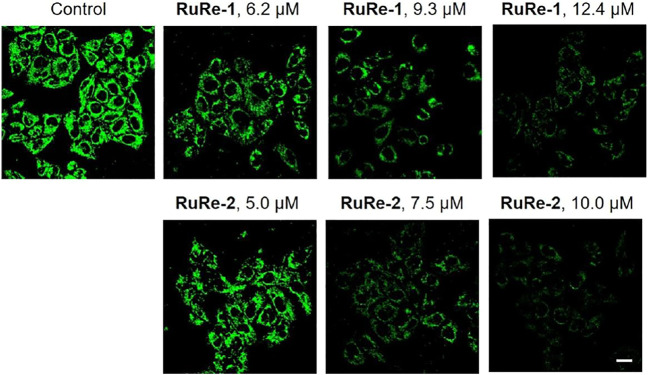
**RuRe-1** and **RuRe-2** induced the loss of MMP analyzed by confocal microscopy with Rh123 staining (*λ*_ex_ = 488 nm, *λ*_em_ = 530 ± 20 nm). The incubation time of the compounds was 6 h. Scale bar: 20 μm.

Apoptosis is associated with a decrease in MMP (Wu et al., 2022), so we investigated the effects of **RuRe-1** and **RuRe-2** on MMP. A decrease in fluorescence intensity of rhodamine 123 (Rh123) can indicate the loss of MMP (Ma et al., 2019). As shown in Figure 6, treated HeLa cells with **RuRe-1** and **RuRe-2** for 6 h induced the decrease in the green fluorescence intensity of Rh123 in a dose-dependent manner. The decline of MMP further confirmed that **RuRe-1** and **RuRe-2** could influence mitochondrial function and promote apoptosis.

**FIGURE 7:**
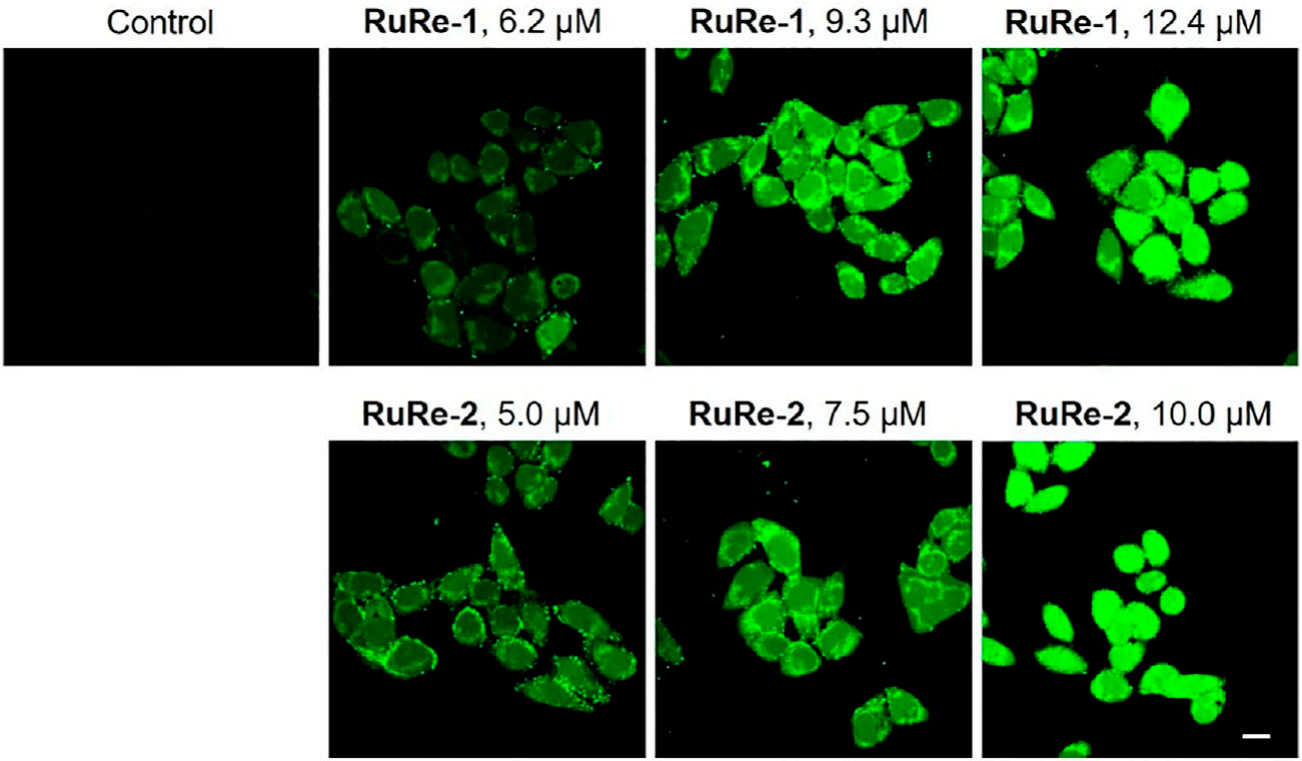
**RuRe-1** and **RuRe-2** induced the elevation of intracellular ROS levels examined by confocal microscopy with H_2_DCFDA staining (*λ*_ex_ = 488 nm, *λ*_em_ = 530 ± 20 nm). The incubation time of the compounds was 6 h. Scale bar: 20 μm.

ROS are natural by-product of normal oxygen metabolism and play an important role in cellular signaling transduction and homeostasis *in vivo* (Casas et al., 2020). It has been shown that the excessive production of ROS may cause oxidative stress and lead to cell death (Jia et al., 2020). Herein, cells were treated with **RuRe-1** and **RuRe-2** for 6 h and then stained with 2′,7′-dichlorodihydrouorescein diacetate (H_2_DCFDA). H_2_DCFDA is non-fluorescent and can be oxidized by intracellular ROS to highly fluorescent 2′,7′-dichlorofluorescein (DCF) (Abdel Hadi et al., 2021). The intensity of green fluorescence can respond to the accumulation of intracellular ROS. As shown in Figure 7, treated HeLa cells with **RuRe-1** and **RuRe-2** for 6 h induced an increase in intracellular ROS levels in a concentration-dependent manner. It reveals that **RuRe-1** and **RuRe-2** possess a strong ability to cause cell oxidative stress.

### Inhibit Cell Migration and Colony Formation

**FIGURE 8:**
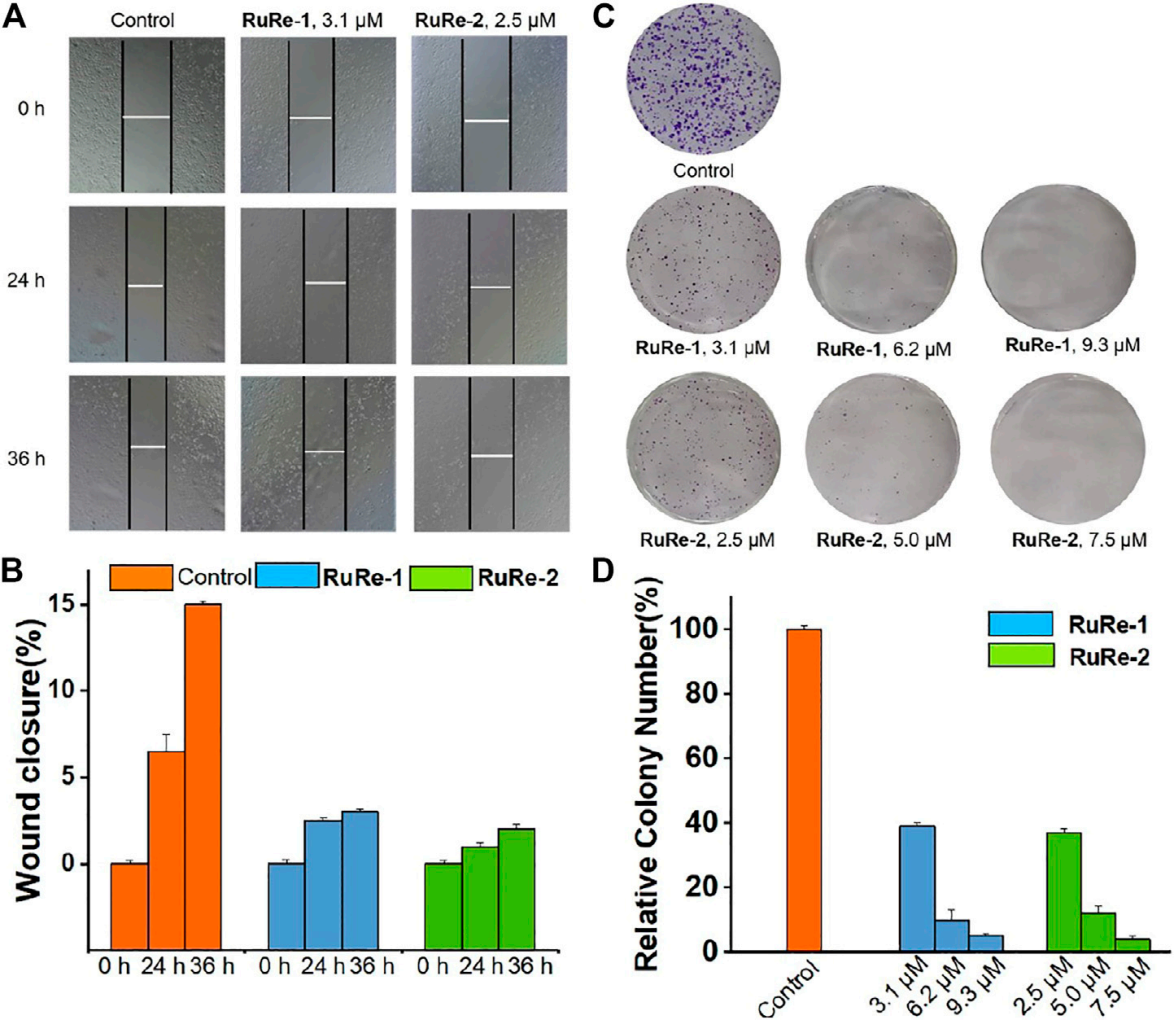
**(A)** Wound healing of HeLa cells after treated with **RuRe-1** (3.1 μM) and **RuRe-2** (2.5 μM) for 0, 24, and 36 h. **(B)** Quantitative data of wound-healing. Wound closure (%) = [1−(width at indicated time)/(width at 0 h)] × 100%. **(C)** Inhibition of colony formation by **RuRe-1** and **RuRe-2**. **(D)** Quantitative data of colony formation assays.

Metastasis is a major obstacle to cancer treatment, which will cause the failure of cancer treatment and the death of patients (Fouani et al., 2017). Cell migration is the main feature of metastasis. Herein, the effects of **RuRe-1** and **RuRe-2** on inhibiting cell migration were studied through wound healing. As compared to control group, HeLa cells treated with **RuRe-1** (3.1 μM) and **RuRe-2** (2.5 μM) exhibited a significant time-dependent inhibition of wound healing integrity (Figure 8A). After 36 h cultivated, both **RuRe-1** and **RuRe-2** inhibited cell migration, and the wound closure rates were 3% and 2%, respectively, which were lower than 15% of the control group (Figure 8B). The results show that these compounds can effectively inhibit cell migration.

The consequence of increased local cell migration is distal invasion, and the clonal growth of distal invasive cells will produce a second tumor (Wang et al., 2017). We further investigated the ability of **RuRe-1** and **RuRe-2** to inhibit the formation of cell colonies by cell colony formation assay. As shown in Figure 8C, the control group formed multiple cell colonies that covered almost the entire study area, while the number of cell colonies in the experimental groups treated with **RuRe-1** and **RuRe-2** decreased with increasing concentration of the administered drug. Notably, cell populations were almost invisible after treatment with **RuRe-1** (9.3 μM) and **RuRe-2** (7.5 μM). The quantitative graphs showed that the cell populations at this time were only 4.8% and 3.9% compared to the control group (Figure 8D). The results show that **RuRe-1** and **RuRe-2** are effective in reducing cell migration and inhibiting colony formation.

## Conclusion

In general, two heteronuclear metal complexes, **RuRe-1** and **RuRe-2**, have been synthesized and their antitumor potential has been developed. Confocal microscopy studies have shown that **RuRe-1** and **RuRe-2** can penetrate cells effectively. Cell viability inhibition assays show that **RuRe-1** and **RuRe-2** are selective killers of tumor cells and exhibit higher toxicity to A549R cells than cisplatin. The mechanistic studies reveal that **RuRe-1** and **RuRe-2** induce MMP depolarization, causing damage to the mitochondria, which in turn increases the intracellular ROS levels and further induces apoptosis. Meanwhile, the process of apoptosis is accompanied by the activation of caspases and the arrest of the cell cycle in S phase. Finally, **RuRe-1** and **RuRe-2** exert potent inhibitory effects on migration and colony formation. Overall, the study of **RuRe-1** and **RuRe-2** provides a basis for the synthesis of heteronuclear metal complexes and their antitumor activity research.

## Materials and Methods

### Materials and Instruments

RuCl_3_·nH_2_O (J&K), bpy (J&K), phen (J&K), DIP (J&K), Re(CO)_5_Cl (Sigma Aldrich), silver trifluoromethanesulfonate (Alfa Aesar), 4-pyridinecarboxaldehyde (Alfa Aesar), 5,6-diamino-1,10-phenanthroline (Alfa Aesar), NH_4_PF_6_ (Alfa Aesar), 4% paraformaldehyde (Beyotime), crystal violet (Beyotime), Dulbecco’s Modified Eagle Medium (DMEM, Gibco), Roswell Park Memorial Institute 1640 (RPMI 1640, Gibco), Fetal bovine serum (FBS, Gibco), penicillin-streptomycin (Gibco), MTT (J&K), Rh123 (J&K), H_2_DCFDA (J&K), Hoechst 33342 (J&K), Annexin V-FITC Apoptosis Detection Kit (Beyotime). Primary antibodies against caspase-3 and PARP were purchased from Cell Signaling Technology. **RuRe-1**, **RuRe-2** were dissolved in DMSO just before the experiments, and the concentration of DMSO in biological experiments was 1% (v/v). Cisplatin was dissolved in 0.9% sodium chloride solution just before use.

A LCQ DECA XP spectrometer was used for obtaining ESI-MS spectra. A Bruker Avance 600 spectrometer was used for obtaining ^1^H NMR spectra. A SpetraMax M2 plate reader was used for determining cell viability. A Nikon A1R/A1 laser-scanning confocal microscope was used for obtaining cell imaging images. A CyFlow Space flow cytometer was used for performing the flow cytometry analysis.

### Synthesis of Heteronuclear Ru(Ⅱ)-Re(Ⅰ) Metal Complexes

Mononuclear complexes **Ru-1** (Halpin et al., 2013) and **Re-1** (Woźna and Kapturkiewicz, 2015) were synthesized according to literature methods. **Ru-2** was prepared following a similar procedure to that of **Ru-1**, except that *cis*-[Ru(phen)_2_Cl_2_]·2H_2_O (Sullivan et al., 1978) was used instead of *cis*-[Ru(bpy)_2_Cl_2_]·2H_2_O (Hartshorn and Barton, 1992).

**Ru-2**: ^1^H NMR (600 MHz, [D_6_]DMSO) *δ* 9.08 (t, *J* = 14.2 Hz, 1H), 8.87 (d, *J* = 5.2 Hz, 1H), 8.78 (d, *J* = 8.2 Hz, 2H), 8.40 (s, 2H), 8.23 (d, *J* = 5.9 Hz, 1H), 8.14 (d, *J* = 5.0 Hz, 1H), 8.06 (dd, *J* = 19.8, 5.0 Hz, 2H), 7.83 (s, 1H), 7.77 (dd, *J* = 8.2, 5.3 Hz, 2H). ESI-MS (CH_3_CN): m/z 758.1345 [M-PF_6_]^+^, 379.5706 [M-2PF_6_]^2+^.

**RuRe-1**: The synthetic route of **RuRe-1** is shown in Scheme 1. A mixture of **Ru-1** (0.140 g, 0.138 mmol) and **Re-1** (0.131 g, 0.166 mmol) were dissolved in 60 ml acetone. After stirred at 329 K for 24 h under nitrogen, the reaction solution was concentrated to 5 ml. A red precipitate was obtained by dropwise addition of saturated NH_4_PF_6_ aqueous solution. Then, the solid was purified by silica gel column chromatography (acetonitrile: water: saturated potassium nitrate, 100:9:1). The PF_6_ salt of **RuRe-1** was again formed by adding saturated NH_4_PF_6_ aqueous solution, and then dried under vacuum. Yield: 79% (red solid). ^1^H NMR (600 MHz, [D_6_]DMSO) *δ* 9.88 (d, *J* = 5.4 Hz, 1H), 8.97–8.71 (m, 4H), 8.31–8.22 (m, 1H), 8.24–8.12 (m, 3H), 8.08 (dd, *J* = 12.3, 4.7 Hz, 1H), 7.99 (s, 1H), 7.83 (d, *J* = 5.3 Hz, 2H), 7.77–7.64 (m, 5H), 7.58 (t, *J* = 4.0 Hz, 2H), 7.32 (t, *J* = 6.6 Hz, 1H). FT-IR (KBr) ν_CO_/cm^−1^: 2032.45, 1914.89. ESI-MS (CH_3_CN): m/z 656.6029 [M-3PF_6_-H]^2+^, 603.0723 [M-Ru(bpy)_2_L-2PF_6_]^+^, 355.5717 [M-Re(DIP)(CO)_3_-3PF_6_]^2+^. Elemental analysis: calcd (%) for C_65_H_43_F_18_N_11_O_3_P_3_ReRu: C, 44.66; H, 2.48; N, 8.81; found: C, 44.56; H, 2.63; N, 8.92.

**RuRe-2**: Complex **RuRe-2** was prepared following a similar procedure to that of **RuRe-1**, except that **Ru-2** was used instead of **Ru-1**. Yield: 82% (red solid). ^1^H NMR (600 MHz, [D_6_]DMSO) *δ* 9.89 (d, *J* = 5.4 Hz, 1H), 8.89 (s, 1H), 8.81–8.68 (m, 1H), 8.39 (s, 1H), 8.31–8.24 (m, 1H), 8.20–8.12 (m, 1H), 8.08 (dd, *J* = 19.4, 4.7 Hz, 1H), 7.95 (s, 1H), 7.79–7.64 (m, 1H). FT-IR (KBr) ν_CO_/cm^−1^: 2031.69, 1918.85. ESI-MS (CH_3_CN): m/z 680.6032 [M-3PF_6_-H]^2+^, 603.0723 [M-Ru(phen)_2_L-2PF_6_]^+^, 379.5722 [M-Re(DIP)(CO)_3_-3PF_6_]^2+^. Elemental analysis: calcd (%) for C_69_H_43_F_18_N_11_O_3_P_3_ReRu: C, 46.14; H, 2.41; N, 8.58; found: C, 46.46; H, 2.66; N, 8.42.

## Data Availability

The original contributions presented in the study are included in the article/Supplementary Material, further inquiries can be directed to the corresponding authors.
