# Supplementary material for: Synthesis, Characterization and Antitumor Mechanism Investigation of Heterometallic Ru(Ⅱ)-Re(Ⅰ) Complexes
Source: Front Chem. 2022 May 27;10:890925. doi: 10.3389/fchem.2022.890925 (PMC9196629; doi:10.3389/fchem.2022.890925)
Supplement: Supplementary file 1 [file DataSheet1.docx]

Supplementary Material

# Supplementary Data

**Methods**

**Stability test.** The stabilities of **RuRe-1** and **RuRe-2** in PBS or HSA were evaluated by analyzing their UV–Vis spectra at 0, 24 and 48 h. First, the stock solution (1 × 10^−5^ M) of complexes in PBS or HSA was prepared, and then divided them into three equal parts, respectively. The first part of the solution was measured by a UV–Vis spectrophotometer immediately. The second and third part of the solution were kept for 24 h and 48 h, respectively, and then monitored using UV–Vis spectrophotometer.

**Cell lines and culture conditions.** HeLa, HepG2, A549, A549R and LO2 cells were purchased from Nanjing KeyGen Biotech., and cultured in DMEM or RPMI 1640 complete medium at 37 ℃ with 5% CO_2_ atmosphere.

**ICP-MS determination.** HeLa cells were incubated in 6-well plates overnight, then the medium was removed and replaced with fresh medium containing **RuRe-1** (20 μM) or **RuRe-2** (20 μM). After 6 h of incubation, the cells were collected, counted then digested with HNO_3_. Finally, ruthenium and rhenium metal elements were detected by ICP-MS.

**In vitro cytotoxicity assay.** The anti-tumor activity of mononuclear complexes **Ru-1**, **Ru-2**, **Re-1** and heteronuclear **RuRe-1** and **RuRe-2** were determined by MTT (5 mg/mL) assay. Cells were placed in 96-well plates and cultured for 24 h at 37 ℃ in a 5% CO_2_ incubator. After replacing the culture medium with fresh medium containing **RuRe-1** or **RuRe-2** at the indicated concentration, cells were cultured for 48 h. 20 μL MTT was added to each well 4 h before the end of the incubation. Subsequently, the liquid in the cell wells was poured out and 150 μL/well DMSO was added to dissolve the MTT-formazan crystals. The absorbance of living cells at 570 nm was detected.

**Hoechst 33342 staining assay.** After HeLa cells were adhered to the confocal dishes, the medium was replaced with a medium containing the indicated concentrations of **RuRe-1** and **RuRe-2**. After incubation for 24 h, the medium was aspirated and the cells were washed three times with PBS, then fixed with 4% paraformaldehyde. Subsequently, cells were labelled 10 mins with Hoechst 33342 (5 μg/mL) and observed immediately under confocal microscope (λ_ex_ = 405 nm, λ_em_ = 460 ± 20 nm).

**Annexin V/PI staining.** After HeLa cells were adhered to the 6-well plates, the medium was replaced with a medium containing the indicated concentrations of **RuRe-1** and **RuRe-2**. Upon incubation for 24 h, cells were collected and then resuspended in 500 μL of binding buffer containing 5 μL annexin V and 10 μL PI. After 10 mins of co-incubation in dark, flow cytometry was used to analyze the cells (λ_ex_ = 488 nm, λ_em_ = 530 ± 20 nm (annexin V) and 620 ± 20 nm (PI)).

**Western blot analysis.** HeLa cells were plated into the 6-well plates, after treating with different doses of **RuRe-1** and **RuRe-2** for 24 h, the cells were collected and lysed. The protein concentration was determined using the BCA assay kit. Equal amounts of protein were ran on SDS-PAGE and then transferred to the PVDF membrane. After 2 h of closure with milk powder, the Caspase-3 and PARP antibodies were incubated onto the membrane overnight, then incubated with secondary antibodies, and finally the images were displayed.

**Cell cycle analysis.** After HeLa cells were adhered to the 6-well plates, the medium was replaced with medium containing different concentrations of **RuRe-1** and **RuRe-2** for 24 h. Cells were collected and fixed for 24 h with pre-cooled 70% ethanol, then washed twice with PBS and finally stained with PI staining solution in a warm bath at 37 ºC for 30 mins. The samples were detected by flow cytometry.

**Measurement of MMP.** After HeLa cells were adhered to the confocal dishes, the medium was replaced with medium containing the indicated concentrations of **RuRe-1** and **RuRe-2** for 6 h. Subsequently, the medium was aspirated and the cells were washed three times with PBS, then stained with Rh123 (1 μg/mL) for 30 mins. After washing with PBS twice, cells were observed under a confocal microscopy (λ_ex_ = 488 nm, λ_em_ = 530 ± 20 nm).

**Measurement of intracellular ROS.** After HeLa cells were adhered to the confocal dishes, the medium was replaced with medium containing the indicated concentrations of **RuRe-1** and **RuRe-2** for 6 h. Subsequently, the medium was aspirated and cells were washed three times with serum-free DMEM. Then, cells were co-incubated with fluorescent dye H_2_DCFDA (10 μM) at 37 °C for 10 mins and washed three times with PBS and visualized by a confocal microscope (λ_ex_ = 488 nm, λ_em_ = 530 ± 20 nm).

**Wound healing assay.** When reaching 80%–90% confluence, the cells were crossed with a 200 μL pipette tip, then photographed under an inverted microscope to record the width of the scratch. After the cells recovered for 1 h, the serum-free medium containing the indicated concentrations of **RuRe-1** and **RuRe-2** were added and incubated for a specific time (24 h, 36 h), meantime, the width of the scratch was recorded.

**Colony forming assay.** HeLa cells were cultured in 6-well plates at a density of 500 cells per well. After cells had adhered, the medium was replaced with medium containing the indicated concentrations of **RuRe-1** and **RuRe-2** for 24 h. Subsequently, the medium was replaced with fresh medium and the cells were cultured for 7 days to form colonies. The colonies formed were fixed with 4% paraformaldehyde, and stained with crystal violet (0.005%). Finally, colonies were calculated and the images were taken using digital camera.

**Statistical analysis.** Biological experiments were repeated at least 3 times and the results were presented as means ± SD.

# Supplementary Figures and Tables

**Supplementary Figure 1.** Synthetic routes of **RuRe-1** and **RuRe-2**.


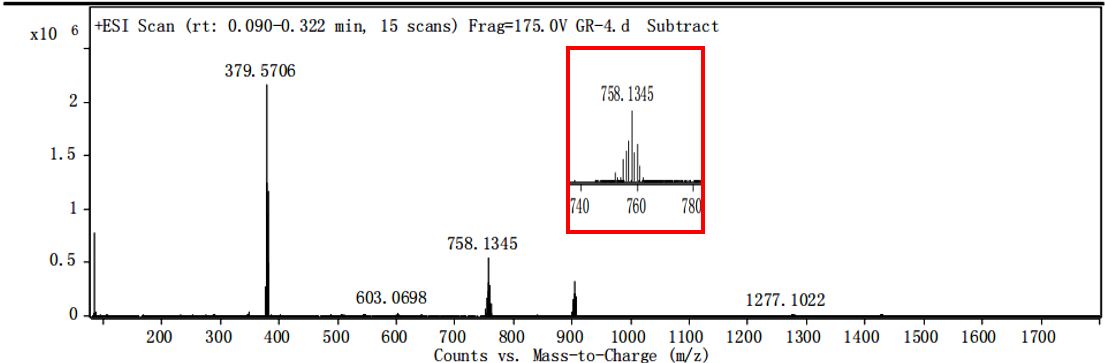


Supplementary Figure 2. ESI-MS characterization of Ru-2, 758.1345 [M-PF_6_]^+^, 379.5706 [M-2PF_6_]^2+^.


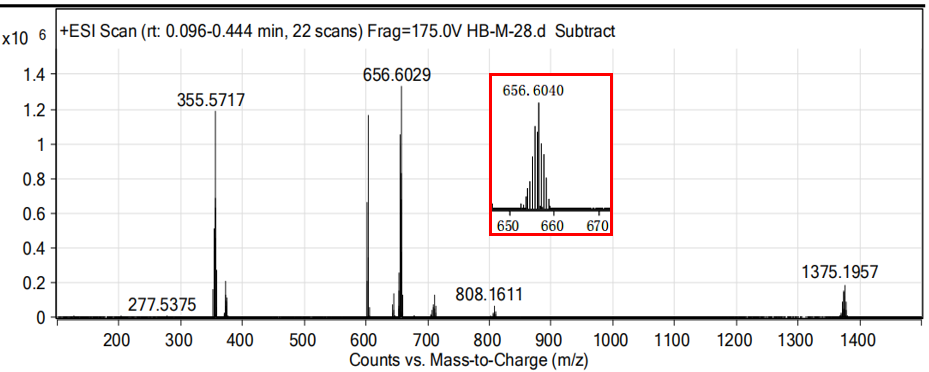


**Supplementary Figure 3.** ESI-MS characterization of **RuRe-1**, 656.6029 [M−3PF_6_-H]^2+^, 603.0723 [M-Ru(bpy)_2_**L**-2PF_6_]^+^, 355.5717 [M-Re(DIP)(CO)_3_-3PF_6_]^2+^.

**
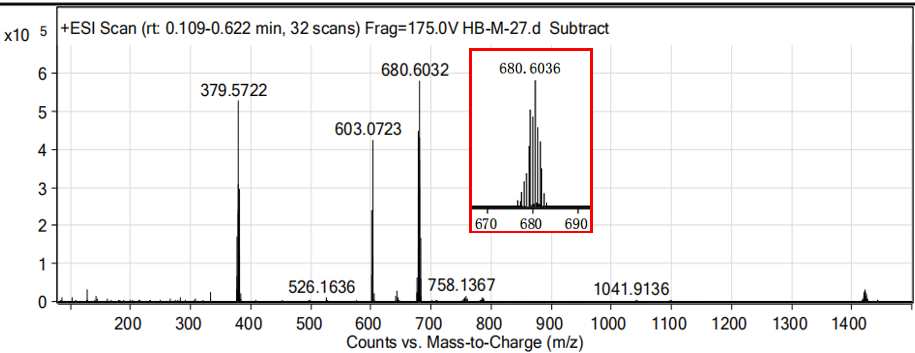
**

**Supplementary Figure 4.** ESI-MS characterization of **RuRe-2**, 680.6032 [M-3PF_6_-H]^2+^, 603.0723 [M-Ru(phen)_2_**L**-2PF_6_]^+^, 379.5722 [M-Re(DIP)(CO)_3_-3PF_6_]^2+^.


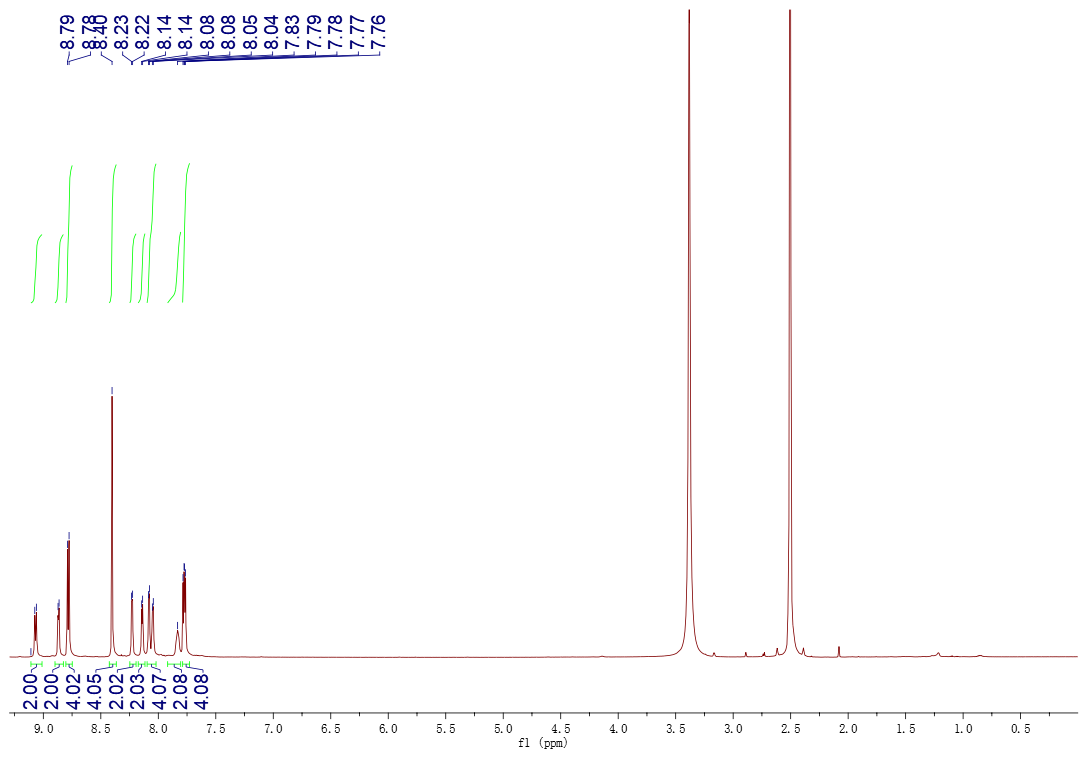


Supplementary Figure 5. ^1^H NMR spectrum of Ru-2 in (CD_3_)_2_SO.


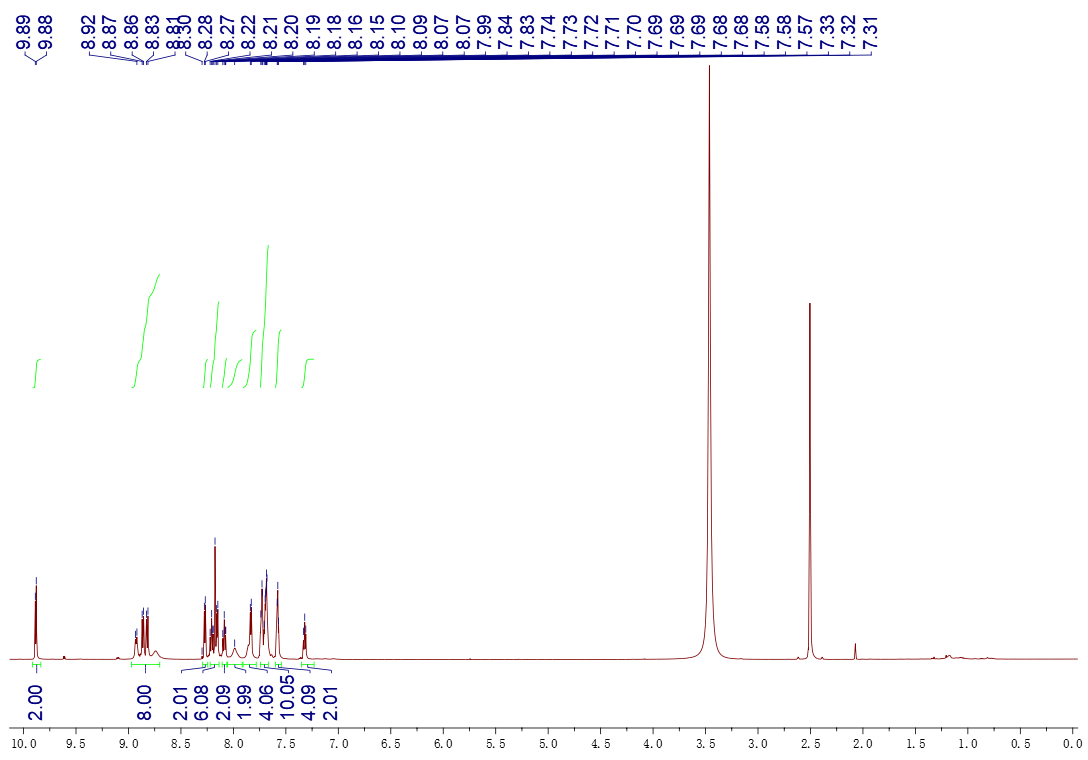


**Supplementary Figure 6.** ^1^H NMR spectrum of **RuRe-1** in (CD_3_)_2_SO.


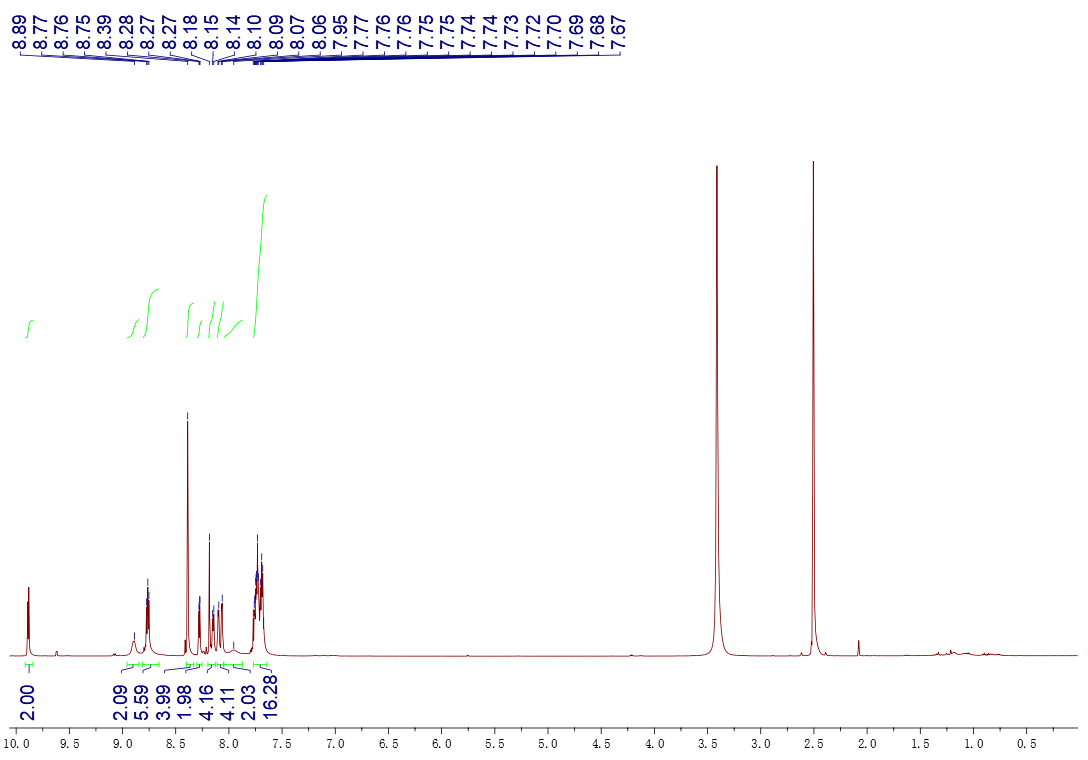


**Supplementary Figure 7.** ^1^H NMR spectrum of **RuRe-2** in (CD_3_)_2_SO.


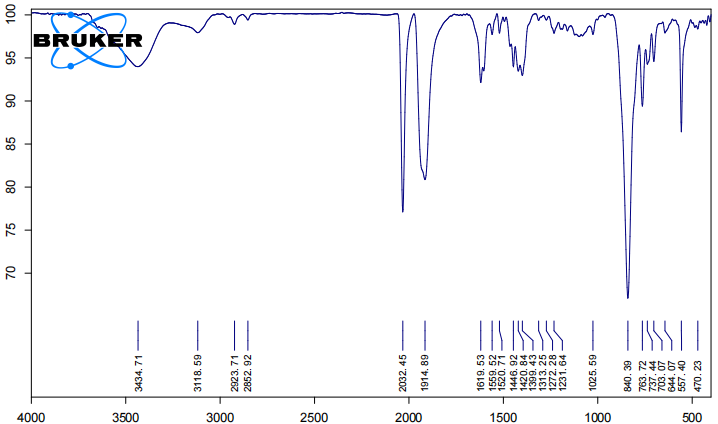


**Supplementary Figure 8.** FT-IR spectrum of **RuRe-1.**


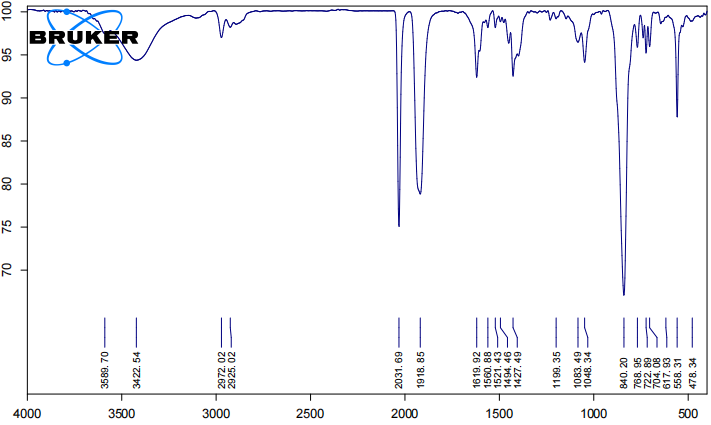


**Supplementary Figure 9.** FT-IR spectrum of **RuRe-2.**


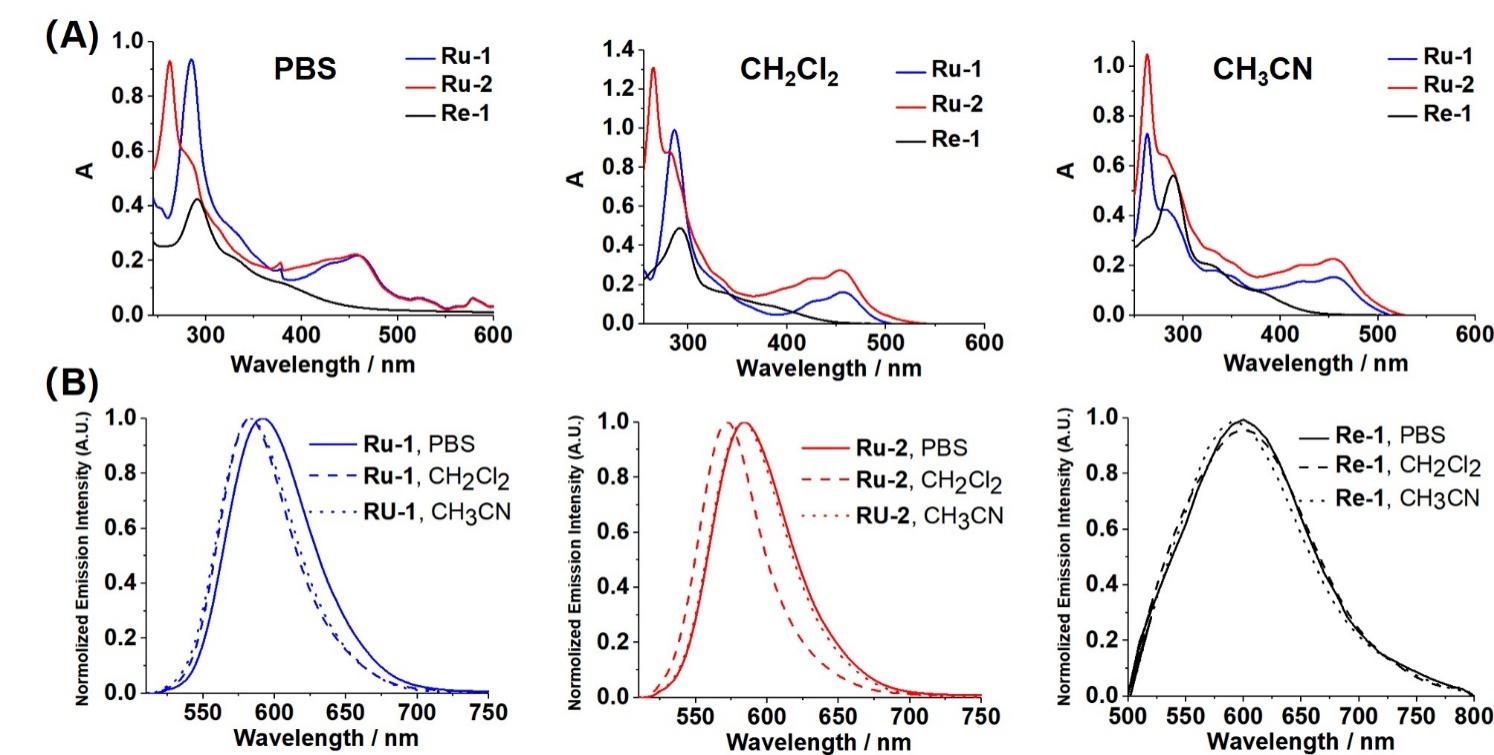


**Supplementary Figure 10.** UV/Vis (A) and emission spectra (B) of **Ru-1** (10 μM), **Ru-2** (10 μM) and **Re-1** (10 μM) measured in PBS, CH_2_Cl_2_ and CH_3_CN at 298 K.


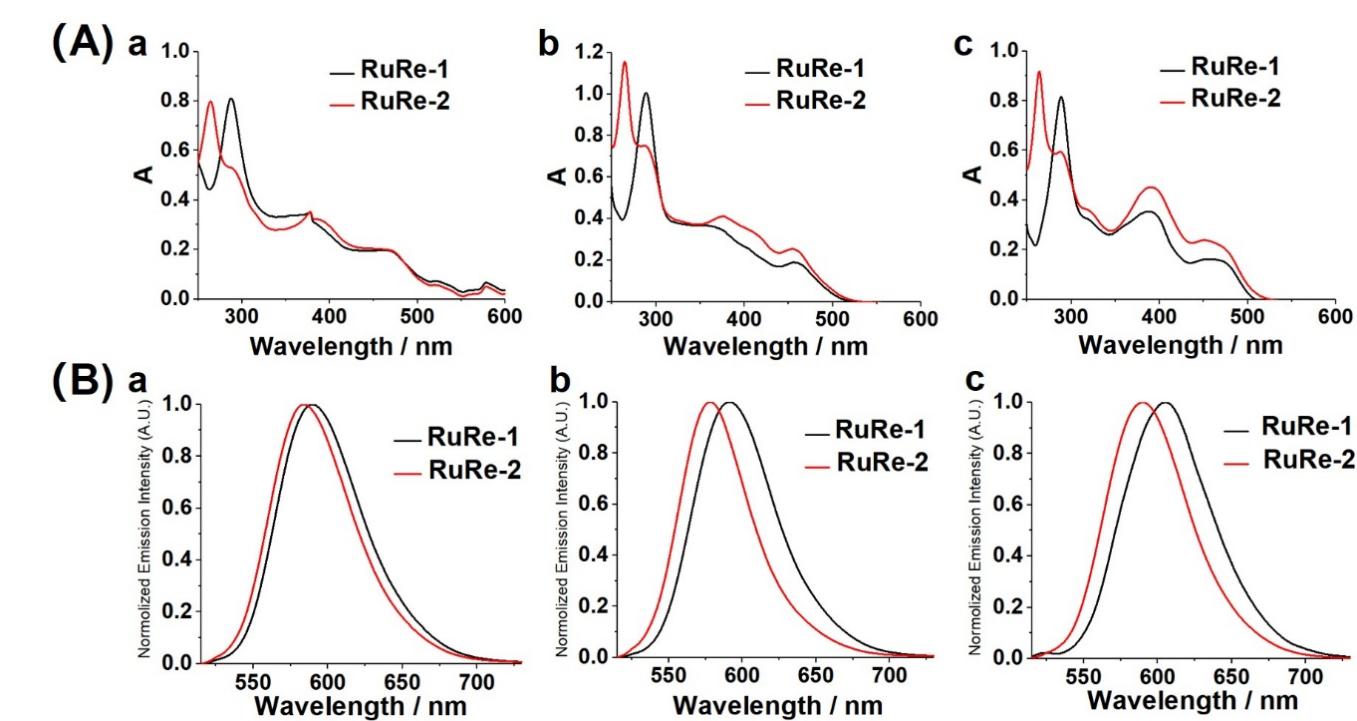


**Supplementary Figure 11.** UV/Vis (A) and emission spectra (B) of **RuRe-1** (10 μM) and **RuRe-2** (10 μM) measured in PBS (a), CH_2_Cl_2_ (b) and CH_3_CN (c) at 298 K.


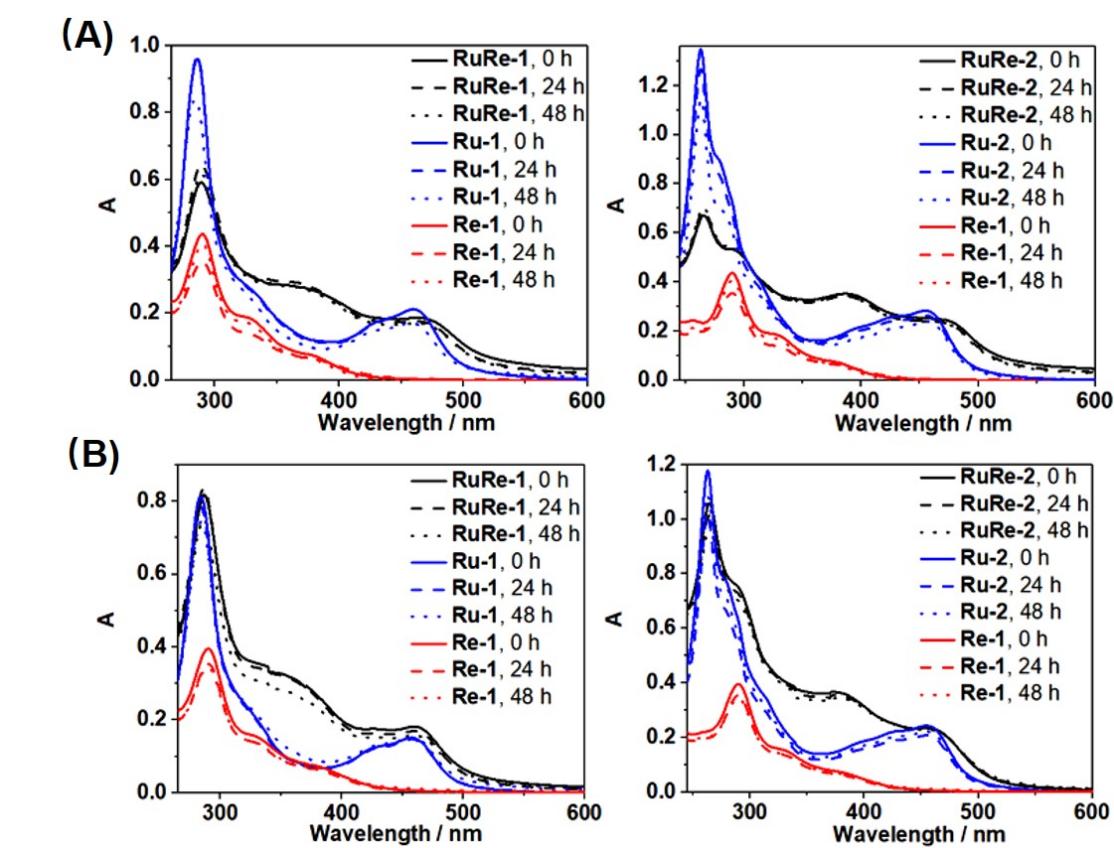


**Supplementary Figure 12.** UV-Vis absorption spectra of **RuRe-1** (10 μM), **RuRe-2** (10 μM), **Ru-1** (10 μM), **Ru-2** (10 μM) and **Re-1** (10 μM) in PBS (A) and HSA (B) solution collected at 0, 24, and 48 h, respectively.


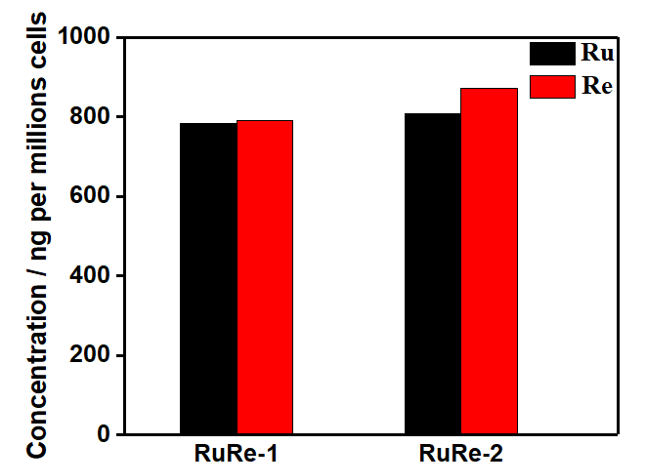


**Supplementary Figure 13.** Quantification intracellular uptake of **RuRe-1** (10 μM, 6 h) and **RuRe-2** (10 μM, 6 h) by ICP-MS.


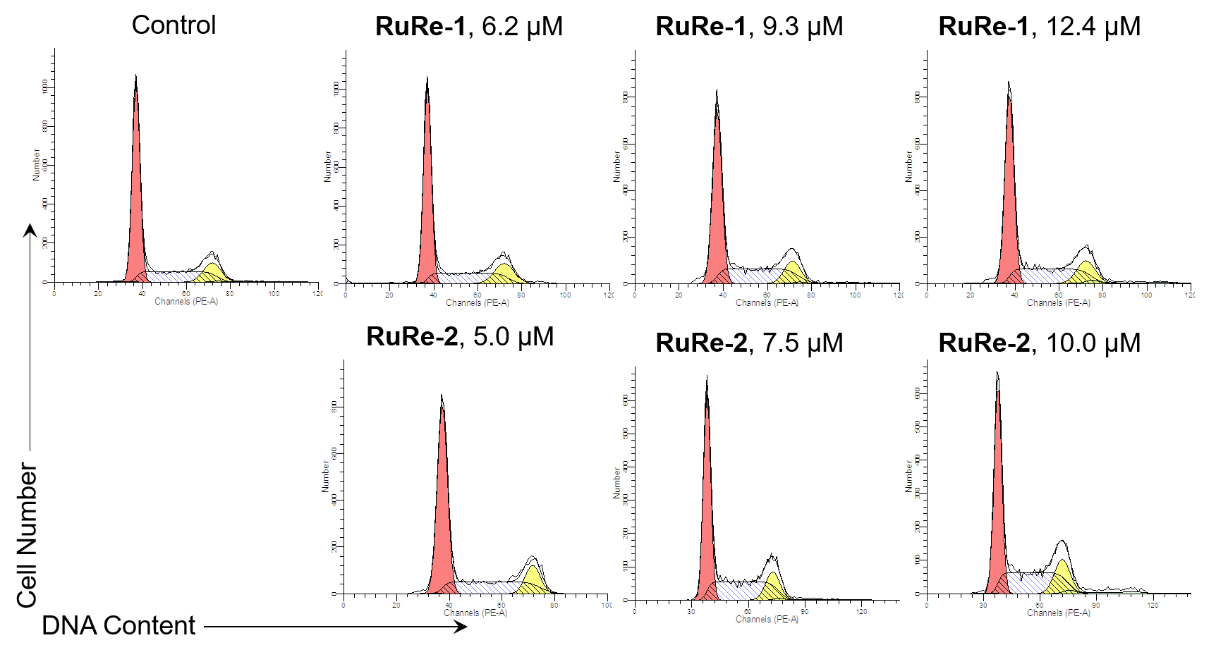


**Supplementary Figure 14.** Effect of **RuRe-1** and **RuRe-2** on the distribution of HeLa cells in cell cycle populations at different concentrations for 24 h treatment.

**Supplementary Table 1.** Photophysical data of **RuRe-1** and **RuRe-2**

| Compounds | Medium | λ_abs_, max (nm) | λ_em_, max (nm) | *Ф*_em_^a^ |
| --- | --- | --- | --- | --- |
| RuRe-1 | PBS | 375 | 590 | 0.042 |
|  | CH_2_Cl_2_ | 360 | 592 | 0.165 |
|  | CH_3_CN | 389 | 605 | 0.116 |
| RuRe-2 | PBS | 374 | 587 | 0.039 |
|  | CH_2_Cl_2_ | 365 | 578 | 0.141 |
|  | CH_3_CN | 392 | 590 | 0.099 |
| Ru-1 | PBS | 458 | 592 | 0.038 |
|  | CH_2_Cl_2_ | 456 | 583 | 0.156 |
|  | CH_3_CN | 455 | 583 | 0.094 |
| Ru-2 | PBS | 456 | 585 | 0.055 |
|  | CH_2_Cl_2_ | 454 | 572 | 0.149 |
|  | CH_3_CN | 454 | 582 | 0.104 |
| Re-1 | PBS | 385 | 602 | 0.124 |
|  | CH_2_Cl_2_ | 392 | 600 | 0.308 |
|  | CH_3_CN | 388 | 600 | 0.215 |

^a^ Solutions of [Ru(bpy)_3_](PF_6_)_2_ were used as the standard, PBS (*Ф*_em_ = 0.042) (Van et al. 1976), CH_3_CN (*Ф*_em_ = 0.062) (Tyson et al. 1999) and CH_2_Cl_2_ (*Ф*_em_ = 0.059) (Pucci et al. 2009) .

**Supplementary Table 2.** Effects of **RuRe-1** and **RuRe-2** on the distribution of HeLa cells in cell cycle populations after 24 h of treatment ^a^

| Compounds | G0/G1 | S | G2/M |
| --- | --- | --- | --- |
| Control | 64.2 ± 1.3 | 14.4 ± 0.9 | 21.4 ± 0.7 |
| **RuRe-1** (6.2 μM) | 68.8 ± 0.5 | 12.5 ± 0.2 | 18.7 ± 1.3 |
| **RuRe-1** (9.3 μM) | 56.5 ± 1.1 | 27.4 ± 0.9 | 16.1 ± 1.1 |
| **RuRe-1** (12.4 μM) | 51.7 ± 0.9 | 33.8 ± 1.2 | 14.5 ± 1.1 |
| **RuRe-2** (5.0 μM) | 65.3 ± 0.9 | 16.8 ± 1.6 | 17.9 ± 1.4 |
| **RuRe-2** (7.5 μM) | 52.7 ± 1.3 | 39.9 ± 1.5 | 7.4 ± 0.5 |
| **RuRe-2** (10.0 μM) | 52.4 ± 1.0 | 40.0 ± 0.6 | 7.6 ± 0.2 |

^a^ Data shown are mean values ± SD of three independent experiments for each treatment.

**Supporting References**

Pucci, D., Bellusci, A., Crispini, A., Ghedini, M., Godbert, N., Szerb, E. I. and Talarico, A. M. (2009). Room temperature columnar mesomorphism and high quantum yield phosphorescence in ionic ruthenium(II) 2,2′-bipyridine-based complexes. *J. Mater. Chem.* 19, 7643-7649. doi: 10.1039/b911017h

Tyson, D. S. and Castellano, F. N. (1999). Intramolecular Singlet and Triplet Energy Transfer in a Ruthenium(II) Diimine Complex Containing Multiple Pyrenyl Chromophores. *J. Phys. Chem. A.* 103, 10955-10960. doi: 10.1021/jp992983w

Van Houten, J and Watts, R. J. (1976). Temperature dependence of the photophysical and photochemical properties of the tris(2,2'-bipyridyl) ruthenium(II) ion in aqueous solution. *J. Am. Chem. Soc.* 98, 4853-4858. doi: [10.1021/ja00432a028](https://doi.org/10.1021/ja00432a028)
